# Heterogeneity prevails: the state of clinical trial data management in Europe - results of a survey of ECRIN centres

**DOI:** 10.1186/1745-6215-11-79

**Published:** 2010-07-21

**Authors:** Wolfgang Kuchinke, Christian Ohmann, Qin Yang, Nader Salas, Jens Lauritsen, Francois Gueyffier, Alan Leizorovicz, Carmen Schade-Brittinger, Michael Wittenberg, Zoltán Voko, Siobhan Gaynor, Margaret Cooney, Peter Doran, Aldo Maggioni, Andrea Lorimer, Ferràn Torres, Gladys McPherson, Jim Charwill, Mats Hellström, Stéphane Lejeune

**Affiliations:** 1 Coordination Centre for Clinical Trials, Heinrich-Heine University Duesseldorf, Duesseldorf, Germany; 2 Copenhagen Trial Unit, Rigshospitalet, Copenhagen, Denmark; 3 Dept. of Biostatistics, Odense University, Odense, Denmark; 4 Centre d'Investigation Clinique, Hôpital Cardiologique Louis Pradel, Lyon, France; 5 Faculté RTH Laennec, Service de Pharmacologie Clinique, Lyon, France; 6 Coordination Centre for Clinical Trials, Philipps Universität, Marburg, Germany; 7 Departments of Health Policy and Health Economics, Eötvös Loránd University, Budapest, Hungary; 8 Irish Clinical Research Infrastructure Network (ICRIN), Dublin, Ireland; 9 General Clinical Research Unit, Misericordiaiae University Hospital, Dublin, Ireland; 10 Centro Studi ANMCO, Firenze, Italy; 11 Hosptital Clinic i Provincial, Barcelona, Spain; 12 Health Services Research Unit, University of Aberdeen, Aberdeen, UK; 13 UK Clinical Research Network Coordinating Centre, Leeds, UK; 14 Department of Oncology, Karolinska University Hospital, Stockholm, Sweden; 15 European Organisation for Research and Treatment of Cancer (EORTC), Bruxelles, Belgium

## Abstract

**Background:**

The use of Clinical Data Management Systems (CDMS) has become essential in clinical trials to handle the increasing amount of data that must be collected and analyzed. With a CDMS trial data are captured at investigator sites with "electronic Case Report Forms". Although more and more of these electronic data management systems are used in academic research centres an overview of CDMS products and of available data management and quality management resources for academic clinical trials in Europe is missing.

**Methods:**

The ECRIN (European Clinical Research Infrastructure Network) data management working group conducted a two-part standardized survey on data management, software tools, and quality management for clinical trials. The questionnaires were answered by nearly 80 centres/units (with an overall response rate of 47% and 43%) from 12 European countries and EORTC.

**Results:**

Our survey shows that about 90% of centres have a CDMS in routine use. Of these CDMS nearly 50% are commercial systems; Open Source solutions don't play a major role. In general, solutions used for clinical data management are very heterogeneous: 20 different commercial CDMS products (7 Open Source solutions) in addition to 17/18 proprietary systems are in use. The most widely employed CDMS products are MACRO™ and Capture System™, followed by solutions that are used in at least 3 centres: eResearch Network™, CleanWeb™, GCP Base™ and SAS™. Although quality management systems for data management are in place in most centres/units, there exist some deficits in the area of system validation.

**Conclusions:**

Because the considerable heterogeneity of data management software solutions may be a hindrance to cooperation based on trial data exchange, standards like CDISC (Clinical Data Interchange Standard Consortium) should be implemented more widely. In a heterogeneous environment the use of data standards can simplify data exchange, increase the quality of data and prepare centres for new developments (e.g. the use of EHR for clinical research). Because data management and the use of electronic data capture systems in clinical trials are characterized by the impact of regulations and guidelines, ethical concerns are discussed. In this context quality management becomes an important part of compliant data management. To address these issues ECRIN will establish certified data centres to support electronic data management and associated compliance needs of clinical trial centres in Europe.

## Background

Clinical research has become impossible without the use of Clinical Data Management Systems (CDMS) to handle the increasing amount of data that must be collected, processed and analysed for clinical trials. In general, trial data are collected at investigator sites with special forms, so-called "Case Report Forms (CRF)", queried, cleaned, stored and analysed with the CDMS. To reduce the possibility of errors, a CDMS employs means to verify the correctness and plausibility of entered data. Another function of CDMS is to code data or to generate reports. The collection of clinical data by means of electronic forms is called Electronic Data Capture (EDC) or Remote Data Entry (RDE). The advantages of using EDC/RDE together with the internet for data management have been recognised early [1-3]. But it did require a long and slow adoption phase in pharma industry and research institutions for the employment of EDC software for clinical trials. Now in nearly half of all trials clinical data are captured electronically [4]. It is generally accepted that EDC/RDE systems can improve the quality of data and accelerate clinical trial conduct [5-7]. Because of restrictions in available funding resources and special requirements for disease specific infrastructures, academic clinical research centres are less likely to implement CDMS solutions that are widely used in pharma industry (e.g. Oracle Clinical™) and often employ smaller and more specialised solutions or develop their own systems. Nonetheless, advanced web-based EDC can be employed very successfully in the academic area [8,9]. For example, with 1500 study investigators and about 22000 randomized patients, INVEST [8] is one of the largest Internet trials based on a web-enabled trial system developed by the University of Florida (USA) including online enrolment, daily patient status reports, records of study visit activities, and calendar and resource management.

The European Clinical Research Infrastructure Network (ECRIN) [10] is an ongoing EU-FP7 funded project to support international academic clinical trials. ECRIN links national networks of clinical research centres (CRC) and clinical trials units (CTU) of 12 national networks and EORTC, and will provide integrated services to investigators and sponsors in multinational studies. To support its trials ECRIN will employ an IT framework, using data management systems located in dedicated ECRIN data centres. As a first step to establish these data centres an overview over the use and the state of CDMS solutions employed by research centres in ECRIN was needed. Therefore, an ECRIN wide survey was carried out to determine the types of CDMS in use, the resources available to the data management units and aspects of quality management and GCP compliance.

The results of our survey show: the kind of infrastructure which is available in centres, what CDMS solutions are in routine use, the state of quality management applied to data management (DM) and the degree of experiences in conducting data management for international clinical trials.

## Methods

**Figure 1:**
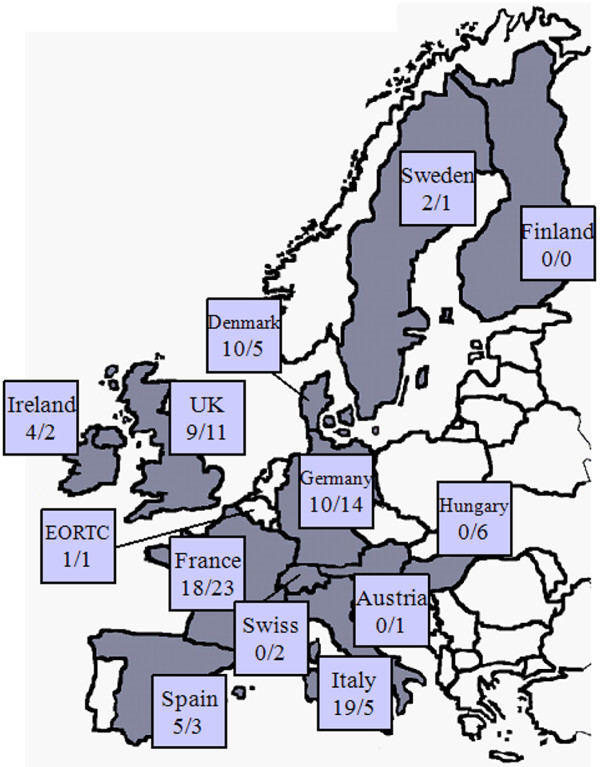
**Participation in data management survey of ECRIN**. Number of participating CTC/CRU of ECRIN partner countries in first survey (N left) and in update survey (N right). Grey: ECRIN member countries.

The ECRIN data management working group conducted a comprehensive standardised survey on data management (DM), software tools and data management procedures for clinical trials with 33 questions. The survey consisted of two parts: a first survey was performed in March/April 2007, which was complemented by a short updated survey in December 2008/January 2009. The second time the new ECRIN members Switzerland and Austria participated in the survey. The questionnaires were sent to 167 ECRIN centres/units (172 in second survey); the response rates were 47%/43% (78 completed questionnaires of the initial survey and 74 completed questionnaires of the updated survey). For Denmark, France, Germany and Italy at least 10 questionnaires were received in the first survey and France, Germany and UK provided at least 10 responses in the second survey (Figure 1).

To conduct the survey we did not apply for a vote of an ethics committee. Ethical approval for the survey was not necessary because first, no patient data were collected and second, the survey involved our partner institutions of ECRIN and was related only to data about DM structures and processes in general and was not connected to a specific clinical trial.

## Results

### Characteristics of the survey

The survey addressed ECRIN clinical research centres and clinical trial units in 12 European countries (Figure 1). With response rates of 47% and 43% the question arises if statements made in our report can be representative for all centres. One has to consider that not all centres that received a questionnaire conduct their own data management. Thus, many centres which didn't use an own CDMS might not have answered the survey at all. Nevertheless, because most questions addressed the percentage of answers in relation to centres with their own DM the answers might still give valuable information about the structure, resources, and software used at these centres. In addition, the response rate of the survey differed considerably between individual countries. In the first survey countries like Denmark, Germany, Italy and Spain, where nearly all centres answered, exhibited a high degree of representativeness. But in countries like France (27%), Ireland (50%), Sweden (11%) and the UK (47%) which showed only a low response rate the representativeness of answers may be rather limited. In an appended question of the survey members were asked about the representativeness of their centre's data management for their country. Even in low response countries, the self-estimated representativeness was relatively high. For example, in the case of UK 5 of 8 (1 missing), France 8 of 12 (6 missing), Ireland 2 of 4, Sweden 1 of 2 (1 don't know) centres declared their DM to be typical and representative for their country. Thus, although the response rate was rather low, the results generated were still valuable. In addition, the value of the answers is strengthened by the fact that answers came from many centres and units that were members of large national clinical research networks (UKCRN, SweCRIN, CIRN, KKS etc.) and therefore often belonged to the leading academic clinical research institutions of their country.

### Characteristics of ECRIN centres that conduct clinical trials

Our survey shows that in general, existing ECRIN centres support all types of trials with a clear focus on trials of phase II (73%) and phase III (85%). 50% of centres also conduct epidemiological trials. A smaller number, but at least one third of centres, carry out phase I trials, surgery trials and medical device trials. The size and the activities of the centres were assessed by the number of ongoing trials and the number of persons employed. Most centres (31%) support less than 10 ongoing trials and employ less than 10 persons (30%); 19% of centres support 10-19 trials. There exist some large centres: 15% of centres support more than 50 trials and 14% of centres employ more than 50 persons. Thus, ECRIN spans a wide range of different sizes of centres/units with a focus on smaller ones.

### Clinical data management systems (CDMS) employed in ECRIN centres

The vast majority of centres/units conduct data management (DM): 64 centres (82% first survey) and 66 centres (89% second survey) (Table 1). In already 61 centres (95% of centres with own DM in first survey) and 55 centres (83% of centres with own DM in second survey) a CDMS is in routine use (Table 1). Different types of CDMS (Table 2) are employed with the focus on commercial products (48%/59%) and proprietary solutions (38%/32%) (Table 2). The share of commercial products seems to have increased during the time between both surveys. Altogether 20 different commercial CDMS products, 7 different Open Source solutions and 17/18 proprietary solutions were identified (Table 3). The fraction of employed Open Source solutions stays relatively small (10%/6%). 79% of the CDMS are own installations of the centre; two centres report access to a CDMS in another unit and two centres have outsourced their entire DM. Thus, outsourcing to external DM centres or other units of an organization/university is still minimal. Of the commercial products the most widely used ones are (first number of first survey, second number of second survey) MACRO™ (14/17) and Capture Systems™ (2/9), followed by solutions that are employed in at least 3 centres: eResearch Network™ (3/3), CleanWeb™ (0/3), GCP Base™ (3/0) and SAS™ (3/3) (Table 4). MACRO™ is a solution that is especially strong with academic customers and is used for example at the Diabetes Trial Unit, University of Oxford, National Blood Service (UK), Institut Gustave Roussy, Paris, Institut Curie, Paris, University of Vienna, several KKS in Germany, Netherlands Cancer Institute. Capture System™ from Clinsight is used in particular by many French investigators.

**Table 1:** Overview of data management

Feature	survey 1		survey 2	
	**N**	%*	**N**	%*

data management performed within centre/unit	64	82	66	89

CDMS system in routine use	61**	95	55	83

Status of data management and CDMS used in ECRIN centres/units. N = number of centres, *% of centres that perform DM. ** includes 4 centres that use a CDMS located in another unit or that have outsourced their CDMS.

**Table 2:** CDMS in use

Category of CDMS	survey 1		survey 2	
	**N**	**%**	**N**	**%**
commercial system	29	48	41	59
open source	6	10	4	6
prorietary	23	38	22	32
others	3	5	2	3

Different types of CDMS in use in ECRIN centres/units. Multiple choices were possible. Total of 61 answers (survey 1), 69 answers (survey 2), % of centres that perform DM.

**Table 3:** CDMS products in use

*Survey 1*	*Survey 2*
**Commercial products**	

MACRO™	MACRO™

eResearch Network™	Capture System™

SAS™	eResearch Network™

Capture System™	CleanWeb™

ECTrial™	SAS™

ClinInfo™	e-MedSolution™

secuTrial™	CITMAS™

ClinTrial™	ClinInfo™

EpiData™*	IBM Lotus Notes™

	Oracle™

	SAS PheedIT™

	secuTrial™

	MS Access™

	InfoPath™

	Teleform™

	WebSpirt™

	SINATRAS™

Number of different commercial products	

9	17

**Open Source product**	

GCP Base™	LAMO Suite™

PhOSCo™	OpenClinica™

PsyGrid™	PhOSCo™

EpiData™	SPAD™

Number of different Open Source products	

4	4

**Self-developed proprietary systems**	

17 different systems	18 different systems

Spectrum of different CDMS products employed in ECRIN centres/units. Multiple choices were possible. Products are mentioned by centres using CDMS routinely. *One questionnaire specified EpiData™ as commercial solution.

**Table 4:** Prevalence of CDMS products

CDMS products	survey 1	survey 2
MACRO™	14	17
eResearch Network™	3	3
Capture System™	2	9
CleanWeb™	0	3
GCP Base™	3	0
SAS™	3	3
ECTrial™	2	0
e-MedSolution™	0	2

Ranking of the most widely employed CDMS solutions in ECRIN centres/units. Number (N) of centres using the product. Total of 61 answers (survey 1) and 69 answers (survey 2), multiple choices were possible. Indicated is the number of times a centre that uses CDMS routinely mentioned a product name for clinical DM.

**Figure 2:**
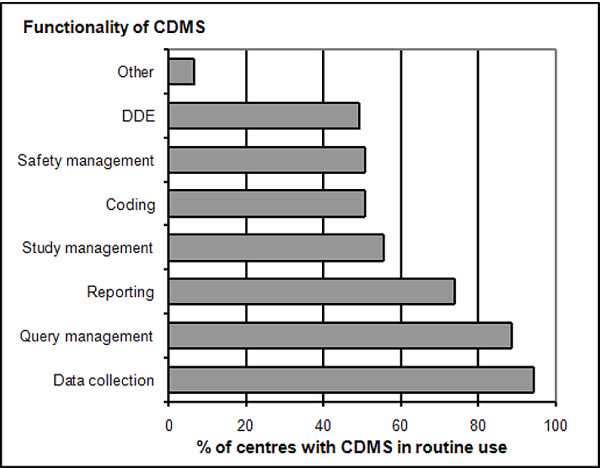
**Extend of functionalities in CDMS**. Implemented functionalities of CDMS used in ECRIN centres (% of centres using CDMS with corresponding functionalities). DDE (double data entry) is the double input of paper based source data into the electronic CRF to avoid type errors.

**Figure 3:**
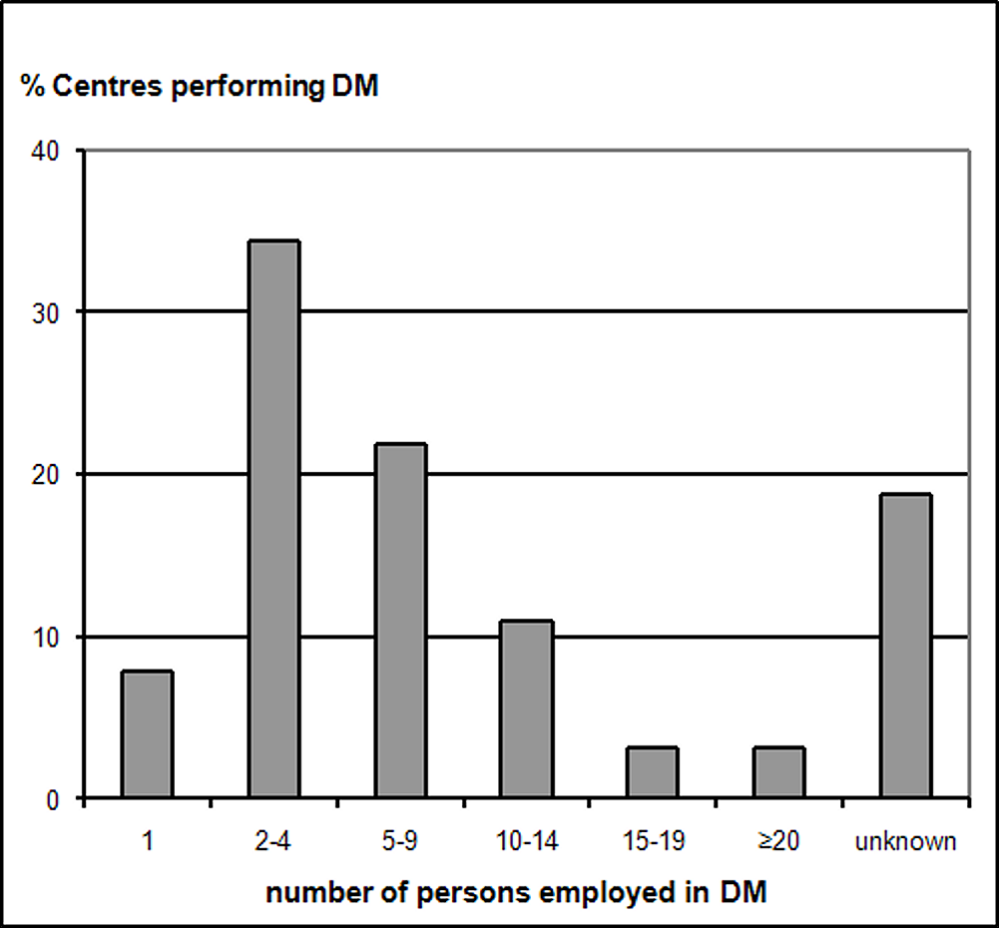
**Human resources available for data management**. Human resources in ECRIN data centres: number of persons employed in DM (% centres performing DM).

Of the different functionalities a CDMS offers the most widely used ones in ECRIN data centres/units are: data collection (94% of centres using CDMS routinely), query management (89%) and reporting (74%) (Figure 2). Double data entry, safety management and study management are supported in approximately 50% of centres with CDMS in routine use. Thus, the focus of the use of CDMS is in data collection and data evaluation, clearly the core functionalities of many centres. About 69% of CDMS (first survey) are using electronic CRFs that allow for data collection at investigator sites (RDE). Online RDE is a major prerequisite for the efficient conduct of multicentre trials, because it allows for centralized data collection. Human resources in ECRIN data centres dedicated to DM are predominantly small and often centres have to manage with only 2-3 persons for their DM (Figure 3).

### Special cases of use of CDMS by ECRIN centres/units

A considerable number of ECRIN centres/units employ special software or use a unique concept for data management in clinical trials:

1. The Copenhagen Trial Unit is using a groupware platform for clinical trial DM. The unit employs Lotus Notes/Domino™ from IBM implemented to meet FDA requirements.

2. CITMAS™ covers patient tracking, enrolment, randomisation, data capture and reporting.

3. JAVA based CleanWEB™ by Telemedicine Technologies is an integrated solution with designer, connector and data collection by web browser. It offers different types of randomisation and on-line monitoring.

4. ClinInfo™, formerly developed for the Clinical Pharmacology Service at University Claude Bernard Lyon, has already been used for international studies of large populations with integrated language options.

5. e-MedSolution™ by International System House Ltd. Budapest is a health care information system and is used by the Hungarian centres for clinical trials.

6. ECTrial™ is a clinical data base software.

7. SINATRAS™ is the EDC system developed by SAKK for the CTU in Bern (Switzerland).

8. Some centres are using data analysis software (SAS™, SPAD™ by Decisia) or database software (MS Access™) for their DM processes.

9. TeleForm™ (Electric Paper) enables the collection of data from different kinds of forms (paper, HTML, PDF) and consists of modules to design forms, scan forms, read, verify and export data.

10. SAS PheedIT™ is a web- and SAS-based solution with modules for study set-up, data entry, report generation, validation management and data export.

11. The data mining software SPAD™, a suite for exploratory and predictive analysis, is also used as a CDMS. SPAD™ consists of data editor, data management, export and transformation functions.

12. One centre is using MS InfoPath™, a tool to create XML-based forms. It can be used in connection with Windows SharePoint™ Services.

Open Source solutions for clinical data management are of special interest in the academic community. For example, PsyGrid™ was initially developed for the DM of large trials of complex interventions in mental health and has been further developed to be used for all sorts of trials. The PsyGrid™ system has been renamed openCDMS™ (since Sep. 2008) and the software is now available under a free licence (LGPLv3). openCDMS™ is used amongst others by the UK Mental Health Research Network, the UK Diabetes Research Network and the National Institute of Health Research (UK). The system is actively developed and maintained by a team of developers at the University of Manchester (UK). The modular web service architecture of the system enables interoperability and even includes online randomisation with email and SMS notification and reporting for project management. PhOSCo™ is in use for several years and was mentioned in both surveys. It uses XML for clinical data and metadata. GCP BASE™ is a web-based tool for remote data capture for clinical trials developed at the Mario Negri Institute for Pharmacological Research in Italy. It is released as free software under the General Public Licence. EpiData™ consists of different modules: "Entry" can be used for simple or programmed data entry and data documentation and "Analysis" performs basic statistical analysis, graphs, and data management.

### Quality management of data management in ECRIN centres

**Figure 4:**
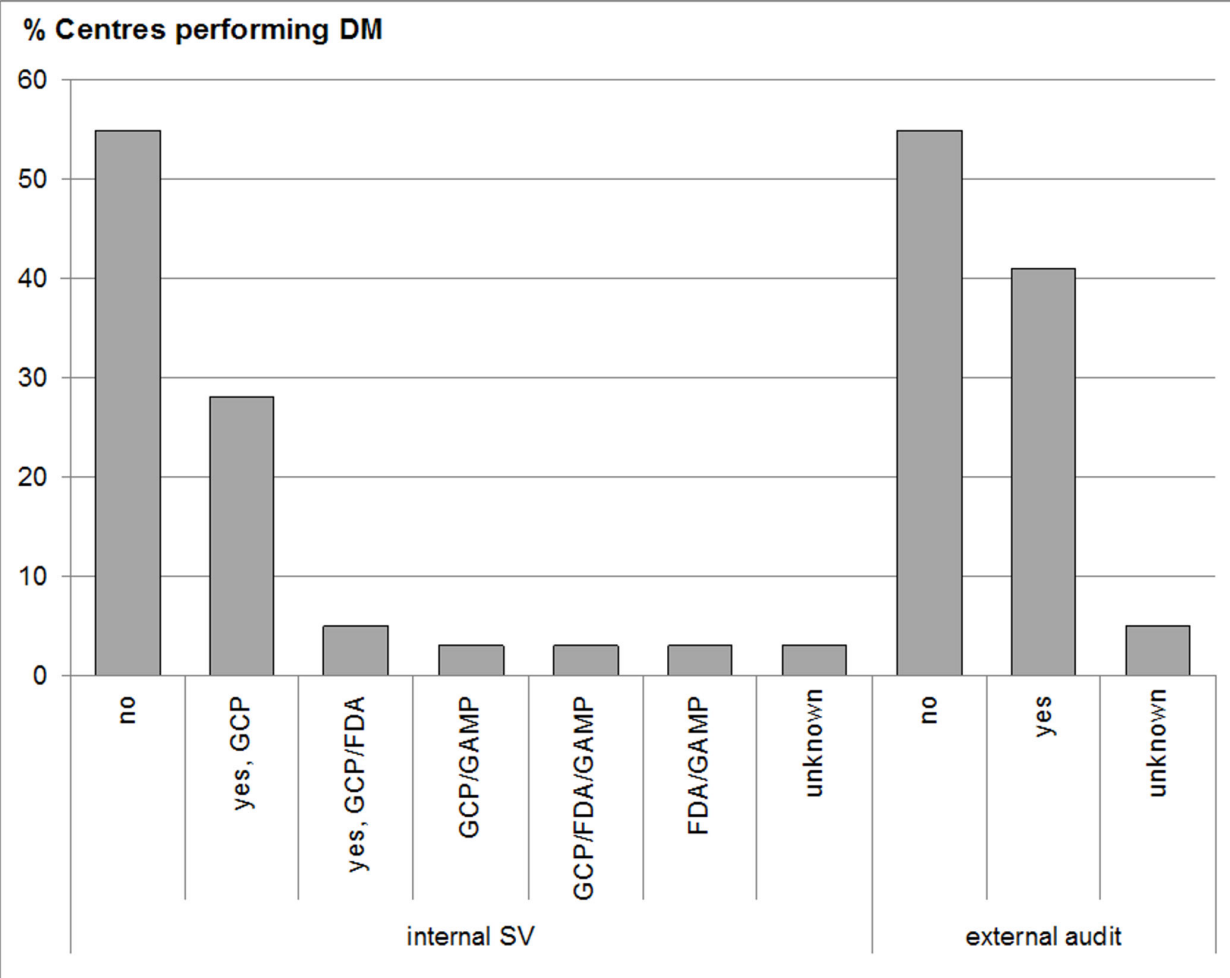
**Quality management of data management**. Availability of CDMS which comply with guidelines and legal requirements: left: DM which is compliant with GCP, GAMP and FDA requirements (internal system validation) and right: DM which has undergone independent validation (external audit). SV: system validation, GAMP: Good Automated Manufacturing Practice, FDA: Food and Drug Administration (here: 21 CFR Part 11), (shown are % centres performing DM).

In the area of GCP compliant clinical trials, a quality system must complement clinical data management operations, to protect patients and to ensure that the collected data are correct. This quality system prescribes for example independent audits to determine whether DM activities are conducted correctly according to study protocols, standard operating procedures (SOPs), and GCP. A quality management system for data management is in place in 64 (91%) centres/units performing DM (first survey); 50 centres/units (78%) have data management SOPs available. Although quality is ensured by nearly all centres/units, over half of the centres still need to conduct complete system validation of their DM (Figure 4). Only about one third of centres validated their DM according to GCP and only 2 centres (3%) performing DM have conducted full system validation covering GCP, FDA (electronic documents) and GAMP. On the other hand, independent, external audits have been performed in already 26 centres (41%) with DM (Figure 4).

64 centres (82%) declared themselves able to provide infrastructure and human resources to support multinational trials. Experience with DM in international trials exists in 47 (60%) centres and 44 (56%) centres have reported to possess a CDMS suited for multinational trials.

## Discussion

### ECRIN data centres

Our survey shows that a majority of ECRIN centres/units is conducting DM and employs a CDMS. About half the centres use a commercial CDMS. But the realisation of DM for efficient and GCP compliant international clinical trials is still a challenging venture for academic research centres. To simplify this undertaking ECRIN will create dedicated and certified "ECRIN Data Centres" to provide DM services for international trials. For this purpose ECRIN will use resources already available in ECRIN centres, instead of building own solutions or buying new applications. Therefore, our survey was supposed to give an overview of DM resources and software solutions available in ECRIN and help to create an inventory. Many of the listed CDMS solutions have already been in use for many trials and ECRIN members have gathered experience using them. But it must be considered, that solutions used in ECRIN might not be optimal for the support of international trials, which have to be GCP-compliant as well as multilingual.

### Heterogeneity of clinical data management solutions

Our survey covers many large European countries and some of the smaller ones (Figure 1). To our knowledge, this is the first time that an overview is published on CDMS tools, DM resources and quality management procedures within such a large number of academic clinical centres in Europe. In contrast, most surveys published have been concerned only with the proportion of electronic data capture in relation to paper based data collection. Recently the number of clinical trials conducted with electronic data capture has been increased considerably. A survey showed that between 2008 and 2009 45% of respondents already conducted more than 50% of their trials using EDC [11]. A recent analysis of clinical trials in Canada showed that the adoption of EDC systems in clinical trials is higher than indicated in the literature [12]: it was estimated that 41% of clinical trials were using an EDC system. Though, trials funded by academic institutions and government were less likely to use an EDC system compared to those sponsored by industry.

Clearly, electronic data capture has reached academic clinical research centres. Our survey shows that the DM situation in European clinical centres and the CDMS in use are surprisingly heterogeneous. In fact, the number of different solution seems to have increased between 2008 and 2009 (Table 3). Not only is no single CDMS product in predominant use, but also different concepts are used for DM (e.g. groupware systems, clinical information systems, data analysis systems are used for study data collection). A large part of clinical centres uses their own developed solution or a single solution not used by any other centre. Open Source CDMS may be an alternative [13], but have not yet been introduced on a large scale so far. Only about 10% of centres use an Open Source solution including GCP BASE™, PhOSCo™, openCDMS™ (PsyGrid™) and EpiData™. OpenClinica™ an Open Source solution which has recently gained in popularity is only used by a single centre. Nonetheless, we know of several centres that have installed OpenClinica™ in addition to their routine system for evaluation.

Although many different CDMS are in use, solutions which are represented strongly in pharma industry (e.g. Medidata™, PhaseForward™, and Oracle Clinical™) were not mentioned. On the other hand, our survey covers many CDMS which have never been mentioned in previous surveys or market analyses [14-16]. A reason for this may be that many vendors of commercial solutions seem to focus for the most part on clients in pharma industry with their considerable financial resources and offer features, like a product or active substance level, that are not useful in academic trials. In addition, commercial software may be disadvantageous for academic research mainly because of high costs and because of risks regarding delivery and future software maintenance. Furthermore, software licences should not restrict the integration with other systems and the upgrading of the IT infrastructure of academic centres. A reason for the high degree of heterogeneity may be the necessity to use specific CDMS solutions for disease specific networks (e.g. cancer, paediatrics, and psychiatrics). For academic trials the shortening of the time to market for a product cannot be the main objective for using a CDMS. For academic clinical trials it is data quality, better recruitment and a more efficient trial conduct that counts. The prime importance of data quality for academic trials is illustrated by the fact, that data analysis software and even data mining solutions are used as CDMS (e.g. SAS™, PheedIT™, SPAD™). Because ECRIN data centres will support European international clinical trials, the considerable heterogeneity in their available DM resources and employed solutions may turn out to be a hindrance for international cooperation and trial data exchange. This may indicate the necessity to further the implementation of common data standards like CDISC (Clinical Data Interchange Standard Consortium) in ECRIN.

The use of CDMS in clinical trials are characterised by the impact of regulations on DM (e.g. 21 CRF Part 11, EU GMP Guideline Vol. 4, Annex 11 Computerized Systems, GCP, data protection laws, e-signature requirements). For clinical centres employing CDMS it becomes necessary to implement best practices for CRF design, query resolution, and study start-up in an EDC environment, including user acceptance testing, system validation, creation of a data management plan and training of investigators in the use of the application. These requirements may cause considerable pressure on the DM resources of a data centre. To provide the necessary quality ECRIN data centres have to accomplish best practices with rather limited resources. Because the most important factors for the quality of clinical trial conduct are good clinical project management together with efficient clinical data management, clinical trial operations depend increasingly on the quality of the IT infrastructure. To conduct international clinical trials IT-based collaborative support can become even more useful because it enables remote monitoring, adverse event notification, and remote review of clinical operations across international sites and various time zones. But our survey shows that most centres are focused only on the core functions of DM (data collection, query management, reporting). This limitation may be necessary in light of the limited resources available in ECRIN data centres, but there is also the necessity to improve quality management by harmonisation. Standards with respect to DM should be promoted in all national networks of clinical trial units and clinical research centres and resources for common quality management should be exchanged. Because of the heterogeneity in CDMS the support of data exchange standards (CDISC, HL7, IHE) becomes a necessity for cooperation in international clinical trials. Therefore, ECRIN centres should try to jointly use DM resources and enforce the support of CDISC standards.

Although highly sophisticated, some web-based EDC systems still seem to show impairments [17], notably that systems are not robust enough to handle the workload (e.g. slow web page refreshing rate), that EDC systems support different versions of basic software (e.g. internet browsers) and that CRF pages are not displayed correctly (e.g. missing data field boxes). In addition, EDC systems often may generate unnecessary queries. To avoid these problems, quality management, best practices and training will be important aspects of DM services of ECRIN data centres. Our survey shows that human resources in many ECRIN centres are often small and that some deficits in quality management exist, especially in the use of system validated CDMS and the documented evidence for data management audits. For this reason the ECRIN working group will design and implement an independent certification process for ECRIN centres to certify centres that are qualified to conduct GCP compliant international trials [18]. To receive a certificate evidence for comprehensive quality management will be of utmost importance and available resources and workload will be critical factors.

ECRIN centres should prepare themselves for new developments in the area of clinical trial DM. Our survey shows that to a large degree only basic functionalities of CDMS are supported. But in future, interfaces between clinics, medical practices, and laboratories, as well as integration with site management, electronic patient reported outcomes (ePRO) and data warehouses will become necessary components. Especially, electronic health records (EHR) will be used increasingly for data collection in clinical trials; because the EHR does not interrupt the investigator's workflow in a way the EDC system does [19]. There will be the need to have CDMS available in ECRIN data centres that will support such EHR based data collection in the future. Therefore, in the heterogeneous CDMS environment of ECRIN the dedicated ECRIN data centres should encourage the use of clinical research standards that allow for interoperability with the EHR (e.g. CDISC healthcare link). This may even contribute to the streamlining of clinical research and therefore improve healthcare for patients.

For the implementation and the use of a CDMS an ethical approval is in general not necessary. Nonetheless, because clinical data management handles and processes human data, it must comply with high ethical standards. The ethical aspects are covered by the need for GCP compliance of any CDMS that is used in a clinical trial. GCP is a standard for design, conduct, performance, monitoring, auditing, recording, analyses, and reporting of clinical trials. Implementation of GCP not only provides assurance that trial data are credible and accurate, but also that the rights, integrity, and confidentiality of trial subjects are protected [20]. In topic 5.5.3 GCP regulates details of using electronic trial data and electronic trial data systems (e.g. audit trail, SOPs, security system, backup). In addition, European Directive 2001/20/EC [21] regulates certain ethical aspects of clinical data management by requiring the existence of safeguards for the rights of trial subjects to privacy and to the protection of their personal data. GCP compliance and data security is guaranteed by a process called "system validation" in which requirements and specifications of CDMS are evaluated. Complete system validation documentation must be produced in case a centre is audited by authorities. In this way, an extensive certification process may support ethical considerations when using a CDMS, ensuing that patient data are collected correctly and used adequately.

## Conclusions

Electronic data capture with professional solutions is already used to a large degree in academic clinical research in Europe. Therefore, ECRIN data centres are well prepared to support international trials. Electronic data capture, data transfer, and web-based systems all can facilitate DM of international clinical trials. ECRIN centres support their DM with a multitude of different CDMS, though the number of trials and human resources are rather small. In addition to dedicated EDC/RDE-systems, several data analysis and data mining solutions are used for DM, illustrating the prime importance of data quality for academic trials. Because ECRIN data centres will support European international clinical trials, the considerable heterogeneity of CDMS may be a hindrance for international cooperation and trial data exchange. This potential problem indicates the necessity to implement in national clinical networks common data standards like CDISC. In a heterogeneous environment the use of data standards can simplify data exchange as well as increase the quality of data. Data management and the use of EDC systems in clinical trials are characterized by the impact of regulations and guidelines. Thereby quality management becomes an important part of compliant DM. To address these issues ECRIN will establish certified data centres to support GCP compliant electronic DM. Because we identified as most important task for clinical research centres to improve their quality management system, especially the validation and GCP compliance of DM, future tasks of ECRIN should be the harmonisation of DM resources for common quality management procedures and self-audits between centres. Based on the results of our survey the ECRIN Working Group on Data Management will develop a certification process for ECRIN data centres, promote the use of CDISC standards in DM, encourage the harmonisation of quality management and system validation, and support international cooperation of clinical DM.

## Abbreviations

CDISC: clinical data interchange standard consortium; CDM: clinical data management; CDMS: clinical data management system; CRF: case report form; CRU: clinical research unit; CTC: clinical trial centre; DDE: double data entry; DM: data management; ECRIN: European clinical research infrastructure network; EDC: electronic data capture; EHR: electronic health record; EORTC: European organisation for research and treatment of cancer; ePRO: electronic patient reported outcome; FDA: federal drug agency; GAMP: good automated manufacturing practice; GCP: good clinical practice; IHE: integrating the healthcare enterprise; INVEST: international Verapamil SR Trandolapril study; IT: information technology; LGPL: GNU Lesser General Public License; RDE: remote data entry; SMS: short message service; SOP: standard operating procedures; SV: system validation; XML: extensible mark-up language.

## Competing interests

The authors declare that they have no competing interests.

## Authors' contributions

WK and CO designed, conducted and analysed the results. The other authors of the "ECRIN Transnational Working Group on Data Management" participated in the development of the survey and discussion of the results. All authors read and approved the final manuscript.
